# Radiotherapy for primary thyroid adenoid cystic carcinoma

**DOI:** 10.1515/biol-2022-0547

**Published:** 2023-03-07

**Authors:** Xiaoyu Duan, Tingting Hu, Hongyi Cai, Lili Lin, Lu Zeng, Huixia Wang, Lei Cao, Xuxia Li

**Affiliations:** The First Clinical Medical College, Gansu University of Chinese Medicine, Number 35 Ding Xing East Road, Lanzhou, 730000 China; Department of Radiotherapy, Gansu Provincial Hospital, Number 204 Dong Gang West Road, Lanzhou, 730000 China

**Keywords:** radiotherapy, primary thyroid adenoid cystic carcinoma, metastasis

## Abstract

Primary thyroid adenoid cystic carcinoma (PTACC) is an extremely rare type of mucin-secreting adenocarcinoma. Currently, it is difficult to diagnose, and it lacks standard treatment protocols. We report the case of a 53-year-old female patient with PTACC who underwent additional intensity-modulated radiotherapy 1 month after surgical treatment with an uneventful course. No invasion or distant metastasis was detected at the 7-month follow-up after radiotherapy, and the prognosis was favorable. In this case, herein, we have summarized the diagnostic features of the disease and proposed that postoperative adjuvant radiotherapy can significantly improve the patient’s prognosis. Finally, we further confirmed the important role of radiotherapy in PTACC by reviewing relevant literature, which may provide clinicians with valuable treatment experience.

## 1 Introduction

Adenoid cystic carcinoma (ACC) is a rare low-grade malignant mucus-secreting adenocarcinoma that accounts for only 1% of head and neck tumors [1]. ACC most commonly occurs in the salivary glands but can also occur in the larynx, trachea, lung, mediastinum, skin, breast, and other rare sites [1–3]. Primary thyroid adenoid cystic carcinoma (PTACC) is extremely rare, and only four cases of PTACC have been reported in China after reviewing domestic and foreign literature. The diagnosis and differential diagnostic features of this disease are not well understood by clinicians, and the treatment options are still controversial. In this study, we report the diagnosis and treatment of a patient with PTACC and analyze the literature on other rare cases of metastasis to the thyroid gland to further clarify the important role of radiotherapy in the treatment of this disease.

## 2 Case presentation

A 53-year-old female patient developed anterior cervical pain in 2021, with intermittent dull pain, no fever, chills, dysphagia, dizziness, palpitation, hand shaking, and no general discomfort, with no other relevant medical history. Color Doppler ultrasonography of the neck and thyroid showed that the lymph nodes on the left side of the neck were visible, the left lobe of the thyroid was hypoechoic, and sub thyroiditis was suspected. The discomfort was relieved slightly after symptomatic treatment.

After 3 months, the above symptoms occurred intermittently, and the patient came to our hospital for additional treatment. On physical examination, a hard mass measuring approximately 1 × 1 cm^2^ was palpable in the left lobe of the thyroid, with a clear boundary, moderate mobility, no tremor, and no obvious vascular murmur. Parathyroid hormone levels: 84.30 pg/mL (normal range 12–88 pg/mL) and thyroid function were normal. Thyroid color Doppler ultrasonography showed that the solid nodules on the left side of the thyroid gland were CTI-RADS4b type, the lymph nodes on the left side of the neck (VI region, V area) were enlarged, and the structure of the lymph nodes was not obviously abnormal. Pathological examination of the left lobe of the thyroid revealed a suspected malignant tumor (SM Bethesda V) and papillary thyroid carcinoma (PTC).

**Figure 1:**
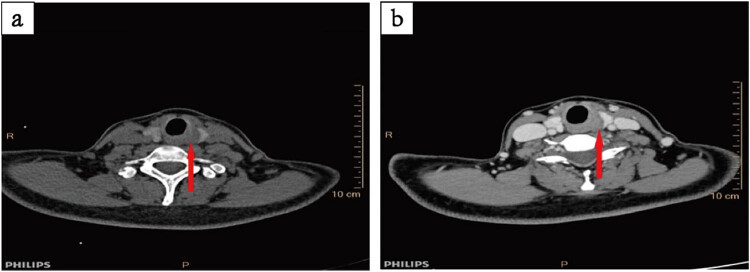
Preoperative soft tissue axial CT scan of the neck (a) + enhancement (b): the left lobe of the thyroid gland shows a hypodense nodule with blurred margins (about 0.6 cm in diameter).

**Figure 2:**
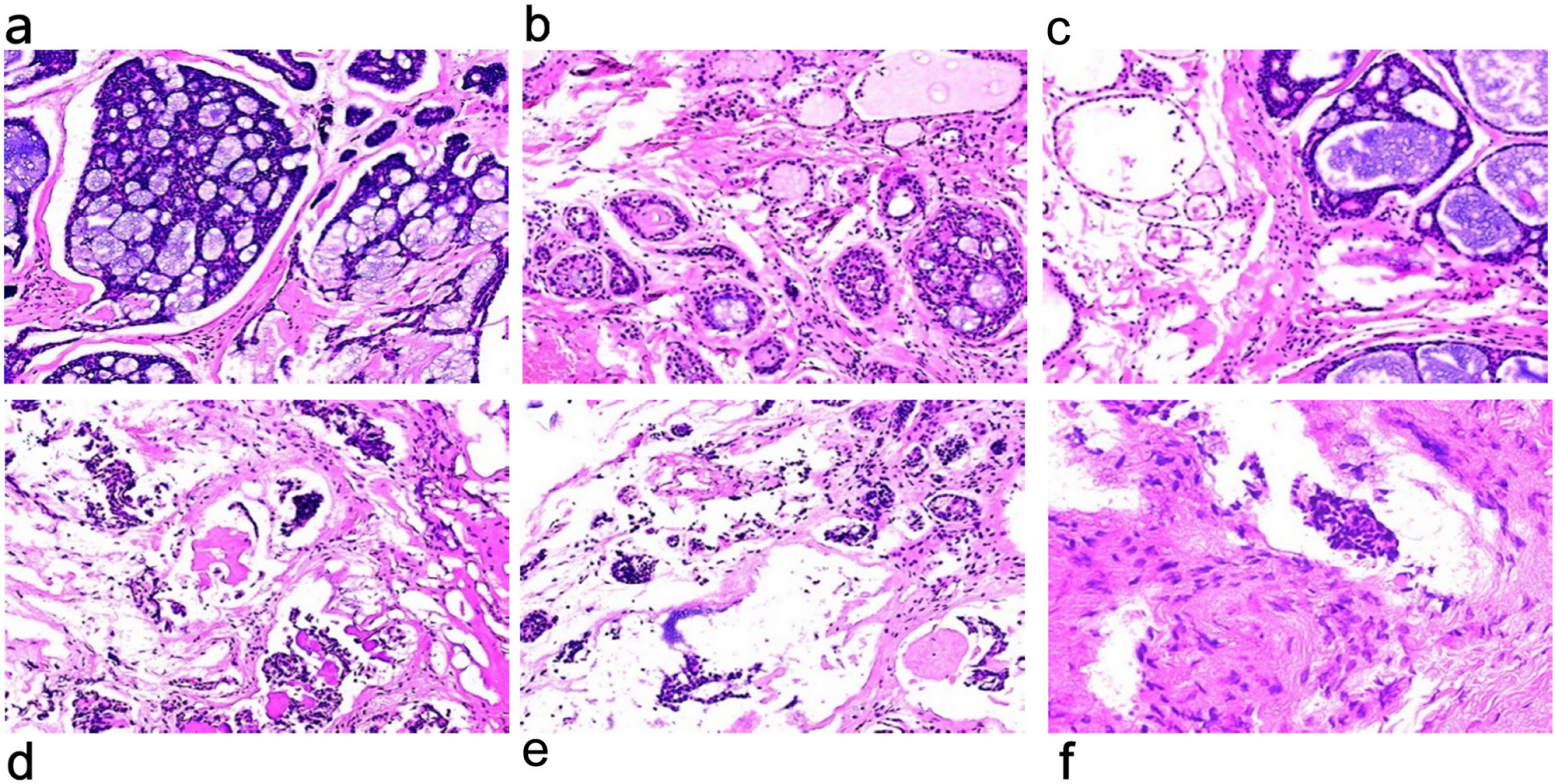
(a–c) Pathological findings of postoperative left thyroid swelling. (d–f) Pathological findings of postoperative thyroid tumor tissue.

Cervical soft tissue computed tomography (CT) showed low-density small nodules in the left lobe of the thyroid, and no obvious abnormalities in the nasopharynx, laryngopharynx, oropharynx, bilateral submandibular gland, and parotid gland (Figure 1). Abdominal ultrasound and chest radiography findings were normal, and no abnormalities were found in the trachea, mediastinum, or other organs. Subsequently, bilateral total thyroidectomy, left central lymph node dissection, and parathyroid transplantation were performed. During the operation, solid nodules approximately 2.0 × 1.8 cm^2^ in size were detected in the left lobe of the thyroid, which included the recurrent laryngeal nerve. Intraoperative frozen pathology of thyroid tumor tissue and left thyroid suggested a thyroid follicular tumor with undetermined malignant potential. The preliminary consideration was follicular subtype papillary carcinoma and medullary carcinoma (MC). After surgery, pathology of the right goiter showed nodular thyroid hyperplasia, and lymph node reactive hyperplasia in the pre-laryngeal lymph node (0/1) and central lymph node (0/3). The pathological and immunohistochemical results of the left thyroid and thyroid tumor showed ACC (Figure 2) as well as the following: 1. P53 (+, missense mutation), 2. Bcl-2 (+), 3. P63 (myoepithelium +), 4. SMA (myoepithelium +), 5. S-100 (−), 6. CEA (−), 7. CD117 (+), 8. Ki-67 (index: 15%), and 9. CK8/18 (+). According to the preoperative cervical CT and related examinations, the possibility of ACC metastasis or invasion of the thyroid gland in other parts was excluded. The patient was diagnosed with PTACC. Levothyroxine sodium tablets were administered for a long time after the operation.

**Figure 3:**
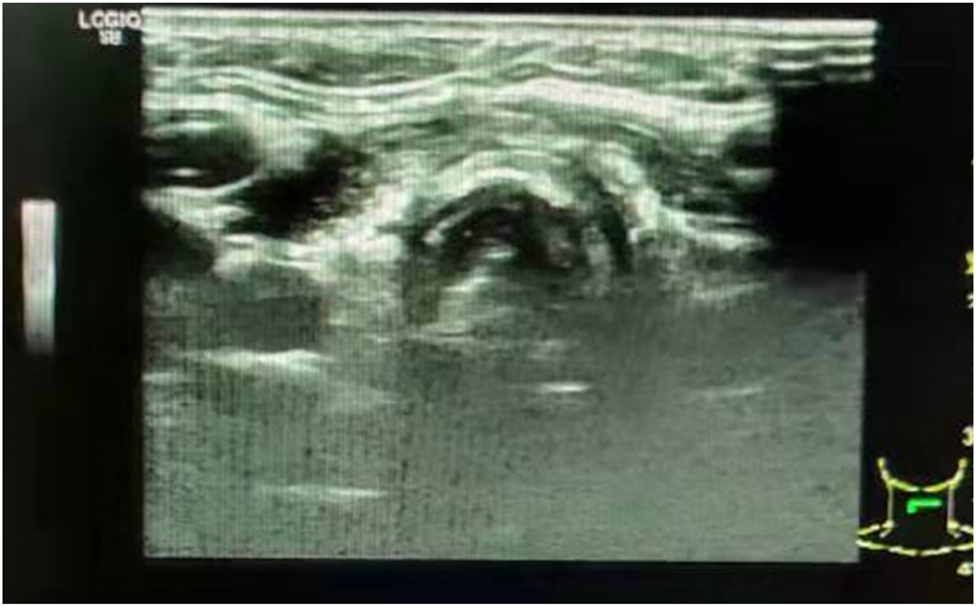
Thyroid color ultrasound: no obvious abnormal echo was observed in the thyroid area.

Because PTACC tumors encapsulate the laryngeal nerve and connect closely during operation, the disease can easily invade the peripheral nerves or form distant metastases. Thus, postoperative adjuvant radiotherapy is recommended to improve patient prognosis. One month after surgery, the patient received three-dimensional conformal intensity-modulated radiotherapy (IMRT) and radiotherapy planning: planning gross tumor volume (PGTV), 69.6 Gy/2.32 Gy/30 f; PTV, 60 Gy/2 Gy/30 f. One month after radiotherapy, the results of thyroid ultrasound showed that there was no abnormality in the thyroid area (Figure 3). Five months after radiotherapy, soft tissue magnetic resonance imaging (MRI) of the neck suggested postoperative changes in bilateral thyroidectomy, and no significant abnormalities were observed in the nasopharynx, laryngopharynx, oropharynx, bilateral submandibular glands, and parotid glands. Multiple small lymph nodes were found in the submandibular area and in the interstitial space of both sides of the neck.


**Informed consent:** Informed consent has been obtained from all individuals included in this study.
**Ethical approval:** The research related to human use has been complied with all the relevant national regulations, institutional policies and in accordance with the tenets of the Helsinki Declaration, and has been approved by the author s institutional review board or equivalent committee.

## 3 Discussion

### 3.1 Diagnosis and differential diagnosis of PTACC

Our review of the literature revealed only four reported cases of PTACC. Of these four patients, three were male and one was of unknown sex, aged 45–59 years, with hoarseness and a neck mass as the main clinical presentation. These patients were finally diagnosed with PTACC based on histopathological and immunohistochemical examinations, after excluding the possibility of other sites as primary tumors [4–7].

The clinical presentation of this patient was intermittent anterior neck pain with no discomfort for the duration of the case. The patient was initially considered to have a subacromial nail infection; however, the treatment was ineffective with intermittent onset of symptoms. The patient’s preoperative pathology was suspected to be PTC, and the intraoperative frozen pathology was suspected to be follicular subtype papillary carcinoma or MC; however, the patient was finally diagnosed with ACC based on the postoperative pathology and immunohistochemistry results. At the same time, according to the neck CT and other auxiliary examinations before the operation, there were no abnormalities in other parts of the nasopharynx, laryngopharynx, mediastinum, and other parts, which ruled out the possibility of primary ACC in other parts. Therefore, the patient was finally diagnosed with PTACC.

A review of other cases of ACC at rare sites shows that the most common feature is slow growth, but extreme susceptibility to recurrence, metastasis, and invasion, thus making the diagnosis extremely difficult [1,8]. Patients often come to the hospital with symptoms such as neck swelling, dyspnea, and dysphagia. Imaging examinations include neck ultrasound, CT, MRI, and positron emission tomography-computed tomography (PET-CT). Neck ultrasound commonly shows neck nodules of different sizes; CT and MRI help to detect local invasion and distant metastasis of tumors, though MRI is more sensitive than CT; PET-CT can be used to assess prognosis and local invasion and distant metastasis of tumors [9]. Previous studies have suggested that the definitive diagnosis of rare-site ACC relies on histopathology and fine needle aspiration cytology (FNAC) examination; however, this claim is also controversial. Some investigators have suggested that FNAC is able to differentiate ACC from interstitial thyroid cancer; Al Khatib et al. were unable to diagnose tracheal ACC using FNAC examination, while in another case, FNAC results were completely different from postoperative pathological findings [10–12]. Therefore, there is still the possibility of misdiagnosis using the FNAC test. The histopathological presentation of PTACC is similar to that of follicular papillary thyroid carcinoma (FPTC) and MC, and immunohistochemical examination is required to confirm the diagnosis. We summarized the immunohistochemical results of five patients with PTACC, including the present case, and found that they mainly showed SMA (+), myoepithelial P63 (+), and Ki-67 proliferation to some extent, with the absence of S-100 (−) and TTF-1 (−). However, patients with FPTC were TG (+), TTF-1 (+), CK19 (+), SMA (−), and P63 (−); thyroid MC characteristically expresses Ctn and usually expresses CEA and RET mutations. Therefore, histopathological examination combined with immunohistochemistry has more confirmatory value than FNAC examinations because immunohistochemistry can identify FPTC and MC by positive expression of SMA, P63, and Ki-67. This is also why we were able to confirm the diagnosis of PTACC.

### 3.2 Treatment of PTACC

There is no standard treatment protocol for this disease, and according to the literature, all four patients underwent surgical resection, and two patients had a good prognosis with no distant metastases and recurrence. The investigators concluded that complete surgical clearance was important and that radiotherapy was not sensitive to the disease [4,6]. One patient developed laryngeal metastases 11 months after surgery, and they concluded that the main reason for the patient’s recurrence was the lack of radiotherapy [7]; one patient underwent postoperative radiotherapy but died of distant metastases within 6 months, and the treatment outcome was poor [5].

In this case, although there was only a single small tumor tissue in the thyroid gland and no evidence of infiltration or invasion into other parts of the body, we found that the recurrent laryngeal nerve was tightly wrapped by the tumor, and it was difficult to remove the lesion completely during surgery to avoid damage to the recurrent laryngeal nerve as much as possible. In addition, considering the fact that PTACC is prone to recurrence and metastasis, we suggest that the patient undergo postoperative radiotherapy. We chose conventional IMRT, and the irradiation field included gross tumor volume (GTV) for imaging visible thyroid lesions. GTV was uniformly expanded by 3 mm to form PGTV. Irradiation dose is referred to as the radiotherapy plan for thyroid cancer. Ultrasonography and MRI of the thyroid gland were performed at 1, 5, and 7 months after the end of radiotherapy, respectively, and no significant abnormalities were found. Based on this case, we believe that radiotherapy has important value in the treatment of PTACC.

To further explore whether radiotherapy can actually achieve good disease control, we summarized all cases of ACC that occurred at other rare sites but eventually manifested as thyroid malignancy by direct invasion or metastasis (Table 1). Their symptoms and diagnoses are very similar to the present disease, which can provide us with clinical experience that can be referred to for better treatment of PTACC. Combined with the analysis in Table 1, all eight patients received surgical treatment, five patients received postoperative radiotherapy, and only one patient received chemotherapy. Postoperative radiotherapy can significantly improve the survival of patients with breast ACC and tongue root ACC [13–15]. Among patients receiving IMRT, proton therapy, and I125 brachytherapy, the latter for recurrent ACC of the head and neck after surgery or external radiation radiotherapy, were also provided, and all of these radiotherapy methods improve patient survival [16–18]. In addition, the investigators concluded that postoperative chemotherapy is not a good option and that targeted therapies such as anti-EGFR monoclonal antibodies and cetuximab may have some impact on patients with advanced ACC [19–21].

**Table 1:** Cases of ACC invasion or metastasis to the thyroid gland in rare sites

References	Sex	Age	Symptoms	Origin location	Size (cm)	TNM stage	Management (thyroid site)	Survival (months)
[1]	F	47	Neck swelling/hoarseness/cough/breathlessness	Trachea	1.7 × 1.6 × 4.4	T4N0M0	RR	>12 (live)
[3]	F	47	Neck lump	The upper anterior mediastinum	10 × 8 × 6	NA	RR + RT + CH	>36 (live)
[11]	F	77	Hoarseness/neck swelling/dyspnoea	Trachea	5.3 × 3.2 × 2.8	pT4N1	RR	>36 (live)
[10]	F	54	Neck swelling/dyspnoea/dysphagia	Trachea	3 × 3	NA	RT	NA
[22]	F	72	Hoarseness	Lung	NA	pT2N1	RR + RT	16 (dead)
[23]	M	46	Dyspnoea/dysphagia	Trachea	NA	NA	LE	1 (dead)
[24]	F	17	Cough/hemoptysis/wheezes/dyspnoea	Trachea	3.4 × 3.7 × 5.0	NA	RR + RT	>60 (live)
[25]	F	44	Neck swelling	Trachea	2.5 × 2.0	NA	LE + RT	NA

## 4 Conclusion

The histopathological features of PTACC are similar to those of FPTC and MC, which are difficult to diagnose and require further differentiation by combined immunohistochemistry, where SMA, p63, and Ki-67 are specifically expressed. Most importantly, previous studies have suggested that radiotherapy is not sensitive to PTACC [22–25]; however, the patient in our case showed good disease control of PTACC with postoperative adjuvant radiotherapy. Combined with a literature review of other rare site ACC diseases, we further confirmed that postoperative adjuvant radiotherapy is of great value in this disease and provides clinicians with experience in its management.
